# Intraoperative manipulation, reduction, realignment, and deformity reduction in patients with atlantoaxial dislocation and basilar invagination

**DOI:** 10.3171/2020.4.FocusVid.20338

**Published:** 2020-07-01

**Authors:** P. Sarat Chandra, Douglas Brockmeyer, Ehud Mendel, Sushil V. Patkar

**Affiliations:** 1 Department of Neurosurgery, All India Institute of Medical Sciences (AIIMS), New Delhi, India;; 2 Department of Neurosurgery, University of Utah, Salt Lake City, Utah;; 3 Department of Neurological Surgery, Ohio State University, Columbus, Ohio; and; 4 Department of Neurosurgery, Poona Hospital and Research Center, Pune, Maharashtra, India

Historically, the treatment for basilar invagination (BI) with atlantoaxial dislocation (AAD; usually irreducible on traction) over the past three decades was a transoral odontoidectomy with posterior instrumented fixation. The occipitoatlantal joint is the center for stability and the atlantoaxial joint is the center for mobility. As the focus shifted to atlantoaxial joints, the treatment strategy also shifted toward the realignment and remodeling of joints. Goel and Harms demonstrated distraction at the C1–2 joints and its fixation. However, this technique has allowed reduction in only one direction, i.e., distraction. In addition, in congenital anomalies, when the C1 is fused with the occiput, there is definitely a greater challenge of placing a screw directly into C1 due to various issues like stability and vertebral artery conflict, as well as more difficulty. This subsequently led to the development of the distraction, compression, extension, and reduction (DCER) technique (Chandra and Agarwal). This allowed a posterior approach only, to reduce, realign, and correct the spinal deformity, leading to a paradigm shift in the management of AAD and BI. In addition, more minimally invasive procedures like endoscopic transoral odontoidectomy evolved to make the transoral procedures less invasive. The following video section on craniovertebral junction specifically deals with techniques that have evolved in the past decade demonstrated by the masters. The techniques presented here range from intra-articular distraction with cage placement (Duan et al.), joint disarticulation techniques for correcting cervical kyphosis (Katzir et al.), mobilization of the vertebral artery while performing C1–2 fixation (Goel), endoscopic endonasal odontoidectomy (Ruiz-Garcia et al.), and navigated transoral odontodectomy (Duan et al.) to more complex procedures, such as C1 lateral mass hypertrophy (Iyer and Brockmeyer), the DCER technique using a specially designed universal reducer (Chandra), use of special VSP plates to reduce atlantoaxial dislocation (Patkar), and an anterior retropharyngeal approach for cage placement and fixation (Patkar). Overall, the varied plethora of cases should not only be of interest for the beginner but also challenge the like of experts. Happy viewing!

